# Is Product Placement Really Worse Than Traditional Commercials? Cognitive Load and Recalling of Advertised Brands

**DOI:** 10.3389/fpsyg.2018.01519

**Published:** 2018-08-20

**Authors:** Tomasz Grzyb, Dariusz Dolinski, Agnieszka Kozłowska

**Affiliations:** Faculty of Psychology, SWPS University of Social Sciences and Humanities, Wrocław, Poland

**Keywords:** advertising, product placement, cognitive load, memory, brand evaluation

## Abstract

Considering the large number of adverts inundating the average consumer every day, the marketing industry is seeking methods to reach clients in a more subtle manner than traditional marketing messages. One such tool is product placement. The article addresses issues of effectiveness of product placement in comparison to a traditional commercial. The objective of the study was to check how participants would recall the content of persuasive messages in conditions of artificially inducted cognitive load (in conditions of traditional advertisement and product placement). Two studies were conducted – a pre-study intended to assess the usefulness of the selected stimuli, and the main experiment. The experiment devoted to this question was planned using an ANOVA scheme 2 (Type of advert: traditional vs. product placement) × 2 (neutral conditions vs. cognitive load). It turned out that the commercial was better retained in neutral conditions than in conditions of cognitive load. The traditional commercial was remembered better than product placement. From the perspective of the study’s ecological validity, of importance is also comparison of retention of brands viewed in a traditional commercial in conditions of cognitive load with retention of brands in conditions of product placement without load. These comparisons indicate the superiority of product placement – brands presented in this manner were more frequently recalled by viewers.

## Introduction

Product placement is a widespread form of promoting products and brands on the contemporary market (Galician, 2013; Marchand et al., 2015). However, the effectiveness of this form of marketing leads to certain questions. For example: is the display of a product or logo for a few seconds during a film comparable with a situation in which the product is directly and clearly displayed during a TV advert lasting half a minute? We start from the premise that during product placement, viewers’ attention is focused on a film, program, weather report, etc., while in the case of a conventional TV advert it is not. As a result, we feel that examining the efficacy of these marketing message presentations to viewers should involve comparison of two conditions: (1) effectiveness of product placement in conditions of attention focused on content and (2) effectiveness of a conventional advert in conditions where such focus is absent. This is the approach we propose in this paper to estimating the effectiveness of product placement.

The field of psychology (applied psychology in particular) has a long tradition of studies on the effectiveness of recall of advertising content transmitted via various media and in various conditions. For example, Frydman (1982) analyzed advertisements published in French magazines, examining their effectiveness by comparing text and visual (graphical) materials. He also examined how the size of persuasive material devoted to exposition of a brand’s logo influenced effectiveness of recall. Of particular importance in Frydman’s studies is how he demonstrated that the application of experimental methods (characteristic of scientific psychology) can serve as an excellent complement to traditional methods applied in marketing. It should, however, be remembered that Frydman conducted his studies primarily in reference to printed advertisements – it would be interesting to explore how his ideas would fare in conditions of television advertisement, particularly considering its significant growth (in comparison to 1982, when he published his paper).

Different countries around the world have different approaches in law to the time that can be devoted to commercials. Under the Australian “Commercial Television Industry Code of Practice” it cannot exceed 13 min in the course of an hour (approximately 22%) between 6 p.m. and 12 p.m., while in the remaining broadcast time it may not exceed 15 min (25%). In the United States, the amount of broadcast time given over to commercials has significantly increased over the last 50 years. For example, in the 1960s a screening of “The Wizard of Oz” took 2 h (with 101 min of time for the film itself); today it lasts 2.5 h or even longer (Freim, 2013). This forces some broadcasters to shorten the film itself to fit all the advertisements in. In turn, a report published in 2013 by the European Broadcasting Union (EBU) shows that within Europe there are significant differences in the amount of TV airtime devoted to commercials. In some Scandinavian countries (Norway, Sweden, Finland) there are no commercials broadcast at all on public television. The same is true in the United Kingdom and Belgium. In turn, Polish public television devotes the largest amount of airtime in Europe to commercials – 18.34%. When combined with time spent on previews of upcoming programming (1.81%) the total exceeds 20%. However, does such a heavy saturation of programming with advertising content lead directly to the success of the companies hawking their products, or at least to their brands being remembered? Studies by Forrester Research on a sample of 588 viewers indicated that 92% of participants declared they regularly avoid watching commercials when they appear on screen, and 30% of them state that they do not watch adverts at all (Zutter, 2005). The real number of viewers potentially reached by an advertising message is estimated at approx. 19%. It turned out that viewers who remained by their TVs during the broadcast of the commercials and did not change the channel generally engaged in other activities at the same time, such as checking their e-mail, responding to text messages, browsing newspapers, talking with someone, or making coffee or tea for themselves (Gunawardena, 2013).

Since the attention of viewers during commercial blocks is transferred to other activities, purchasers of television time are beginning to wonder whether it remains worth investing large amounts of money in the endeavor. As studies conducted by the national association of advertisers in the United States have shown, 78% of them admit that they feel far less certainty and faith in the effectiveness of television commercials than they did 2 years ago. However, they see potential in alternative means of promoting their brands and products, such as sponsoring of products in media, financing of events and advertising films, and product placement (Brown, 1993; Mandese, 2006).

Could a sensible solution from the advertisers’ point of view be product placement? This method of advertising a product or service consists in the intentional insertion of specially selected objects into defined messages, for financial gratification, and with the intention of having a promotional effect (Williams et al., 2011). Advertising a product using this method can be done by highlighting the product or its trademark during selected scenes of a film or TV series, the use of consumption of products by characters in the show, a conversation by characters about the product, or displaying the product’s logo as a background for other events (Corniani, 2001). It is also sometimes the case that product placement is applied in radio broadcasts, and even in literature. Indeed, the first examples of product placement come from the 19th century. One example comes in Jules Vern’s “Around the World in 80 Days,” where the French author places his main character on a ship belonging to a real-life carrier (Gürses and Okan, 2014).

Schudson (1984) submits that the origins of product placement in films can be found in the 1920s, when tobacco companies promoted smoking by having lead actors in films seen smoking cigarettes. In turn, De Beers (the diamond giant founded in 1888) promoted its products in the 1930s and 1940s using product placement, for example by changing the title of the film *Diamonds Are Dangerous* to *Adventures in Diamonds.* De Beers led to *That Uncertain Feeling* including a scene where Merle Oberon can be seen wearing a diamond necklace worth $40,000. In *Skylark* there is a scene involving the selection of a diamond bracelet and brooch for Claudette Colbert – this was also the work of De Beers, and was not included by accident (Segrave, 2004).

Classics of product placement include the somewhat later scenes from *Rebel Without a Cause* in which James Dean fixes his hair using equipment from Ace Comb, which then led to the company achieving greater popularity (Rhee and Rhee, 2015). An exceptionally successful example of product placement came in 1982 with Steven Spielberg’s *E.T.*, in which Reese’s Pieces chocolates were featured, which led to a nearly 66% increase in sales during the first 3 weeks (Dens et al., 2012). Even though product placement is used predominantly for practical reasons (Karrh et al., 2003), the phenomenon is also an interesting area to be investigated in the course of scientific analyses. The history of research in marketing making use of product placement is long enough to provide relatively advanced theoretical models – e.g., the model developed by Balasubramanian et al. (2006). According to these authors, four fundamental elements should be taken into consideration as far as product placement analysis is concerned: execution factors (stimuli based), individual-difference factors, processing type/context/setting and effect(s) of product placement. A similar proposition was made by Russell (1998), who emphasized the role of various cognitive processes. Let us take a closer look at several factors determining the effectiveness of product placement.

Advertising products via the product placement method can occur across many dimensions, with the primary one being the type of product placement in the material. A distinction is made between audio messages, and thus when the name of the brand or product is pronounced by a character on-screen; visual messages, consisting in the appearance of the brand or product itself during a scene; and audiovisual messages, *id est* in which the product is visible on-screen and characters mention it in their dialog (Gürses and Okan, 2014). Studies show that in terms of retention of a product or brand the most effective strategy is an audiovisual message. In accordance with Paivio’s double coding concept (1986), a stimulus activating both audio and visual processes simultaneously strengthens the informational message and eases understanding of it (Clark and Paivio, 1991).

However, the message type most frequently employed by advertisers is the visual message. Interestingly, this is the type of product placement which led among study participants to the worst brand recall, but the most frequently chosen among competing products in a shop (*brand recognition)* (Gürses and Okan, 2014). The division of memory into explicit memory and implicit memory is responsible for this. The former facilitates easy recall of content from the past and experiencing it at the conscious level. However, it does occur that events from the past exert influence on the individual in an entirely unconscious but far more effective manner. This is referred to as implicit memory, which is frequently far more effective than explicit memory.

When speaking of the effectiveness of an advertisement, studies are frequently invoked; however, these are laboratory studies performed in tightly controlled conditions where the participants are encouraged to pay attention when watching commercials (Sharp and Hartnett, 2016). Meanwhile, in natural conditions a commercial break which interrupts a film or show is doubtlessly a sufficient impulse to transfer one’s attention to other activities, for the simple reason that people typically do not like commercials (Blondé and Roozen, 2007). Awareness of this fact has not, however, led to any breakthroughs in the world of marketing research. In spite of viewers’ growing tendency to see commercials in conditions of cognitive multitasking, there remain very few studies examining the effectiveness of advertising messages from this perspective (Duff and Sar, 2015).

The same is true in the study by Blondé and Roozen (2007), which analyses the effectiveness of product placement in comparison to the traditional commercial. An effective advertisement in this case is a combination of proper recall by the viewer of the advertised product and attitude taken toward the brand as a whole. The results of these studies demonstrate that in experimental conditions the traditional commercial is significantly more effective than the product placement method. However, the authors, aware of the phenomenon of cognitive multitasking among viewers that takes place during their daily TV session, added a survey to the study using a 5-point scale to measure the frequency with which they turned their attention to other activities at the moment advertisements appeared on the screen. This allowed for the formulation and confirmation of a new hypothesis according to which, taking into account the results of the survey, the product placement method seems to be a far better marketing tool allowing for more effective recall of the advertised product than the traditional commercial (Blondé and Roozen, 2007). Needless to say, empirical studies aiming at verifying the effectiveness of various forms of product placement have been carried out in the marketing area for some time now. For instance, Gupta and Lord (1998) demonstrated how viewers remember product-related content included in films (in their experiments, the researchers differentiated between prominent and subtle placements). It should, however, be emphasized that there is still an absence of empirical data that would more directly indicate the *de facto* greater effectiveness of product placement over the traditional commercial. Our study aims at filling this gap in the subject literature.

We maintain that in desiring to measure the reactions of consumers to marketing messages similar to those which occur in natural conditions; advertisers should make their best efforts to recreate the everyday conditions viewers typically find themselves in Paas et al. (2003). This is also why we decided to include conditions of cognitive load in the experimental design of our study. This is a very popular approach in product placement studies – e.g., in the study carried out by Pantoja et al. (2016), the researchers also decided to investigate the effectiveness of remembering the content promoted through the use of product placement depending on the cognitive load of the subjects.

In cognitive psychology there are at least two types of metaphors describing the activities of the individual’s cognitive resources. The first and most popular of them is the computer metaphor, which compares the work of the brain to that of a processor which has a limited amount of resources that must be divided among all of the processes in the queue. The second is the energy metaphor, which says that an individual’s cognitive resources can be treated as a specific processing power with definite capacity, facilitating proper performance of a fixed number of operations simultaneously (Gazzaniga, 2014). Both of these approaches show that at a given moment an individual’s cognitive system is capable of processing a limited amount of information, and as a result it should be foreseen that any cognitive effort will lead to its gradual exhaustion.

Let us observe that the above descriptions also apply to the natural situation of an individual sitting in front of the TV. Usually, in the course of watching a commercial break the viewer engages in a range of other activities – checking one’s smartphone for new e-mails, conversing with others in the house, browsing the TV schedule for the coming days (sometimes doing all of these things at the same time). This means that the cognitive resources which can be employed for perception of the commercial break are clearly limited. It would therefore seem that an experimental study can be designed to examine the influence of using a specific advertising stimulus in conditions of artificially induced cognitive load, simulating the natural conditions of receipt of the advertising message. It can thus be assumed that, to the extent a situation involving cognitive load that results from the performance by an individual of some activity at the same time as an advertisement is being shown is a very typical situation for traditional commercials, it is significantly less common in the case of product placement. In the latter situation the individual is generally engaged in actively and purposefully following the action of the film or program on the screen of the TV, cinema or computer. By the same token, if we choose to pose the question of the real effectiveness of product placement compared to the traditional commercial, we should compare situations in which the traditional commercial is viewed in conditions of cognitive load, while product placement is seen in neutral conditions. We therefore decided to conduct an experimental study employing an ANOVA 2 scheme (type of commercial: traditional vs. product placement) × 2 (conditions in which the advert reaches the viewer: neutral vs. cognitive load). Prior to commencing the experiment, it was, however, necessary to prepare materials in a pre-study that would not give rise to methodological doubts. Primarily this means selection of commercials and program fragments for the main study that would not leave any uncertainty as to the brands being advertised.

## Study 1 – Pre-Study

### Materials and Methods

To be sure that the advertised brands in the main study shown in product placements and commercial spots were easily visible for study participants in conditions of focus on the material, two different films were prepared containing both commercials and product placement.

For testing the visibility of logos we used commercials and fragments of films and TV shows containing product placement of such brands as: Evian, Coca-Cola, Gap, Starbucks, Stella Artois, Heineken, Dr Pepper, and Pizza Hut. The study was participated in by 40 individuals divided into two groups. The first watched films consisting of commercials for Evian, Coca-Cola, Gap, and Starbucks as well as product placement of Stella Artois, Heineken, Dr Pepper, and Pizza Hut. The second group watched material with the same brands, but advertised using the opposite mechanism – if the product was advertised to the first group using product placement, in the second it was done so using a traditional commercial spot. After watching the material, study participants were shown a board composed of 88 logos (10 distractors – products from the same category as a product visible in the material – for each brand) and asked to indicate eight of them that appeared in the film. All participants were carefully informed about the whole study procedure and were asked to sign informed consent form before start. The entire procedure (in pre-study and in main experiment) was developed in cooperation with the Institutional Review Board (Komisja Etyki do Spraw Badań) at the Wrocław Faculty of Psychology, SWPS University of Social Sciences and Humanities, and after thorough consideration of the benefits and risks associated with conducting the experiment the Board gave its approval (Decision number 03/E/12/2014).

### Results

On the basis of the obtained results, four brands were identified which were visible by all or nearly all participants (90–100%), and two new films were prepared using them. Material containing product placement was composed of: product placement for the brand Coca-Cola used in the TV series *Breaking Bad*, product placement for the brand Starbucks used in the film *Fight Club*, product placement of the brand Pizza Hut used in the TV series *House of Cards*, and product placement of the brand Dr Pepper used in the film *Spiderman.* The second material contained traditional commercials for these brands, in the same order. A new board was also prepared with logos for measuring the participants’ recall; this time it was composed of 44 brands (analogically, 10 distractors were assigned to each brand of products representing the same category). The materials used in the study are available as an online supplement to this article; they may also be made available on request.

Participants in both the pre-study and the main study were students (and thus young adults with an average age of around 25 years old). This choice was driven by the particular approach of young people to brands and their large influence on purchasing decisions they make (Feltham, 1998).

## Study 2 – Main Experiment

### Materials and Methods

#### Study Participants

The main study included a total of 140 participants – 66 women and 74 men, average age of 25.11 years (*SD* = 7.02). They were sorted randomly into four groups of 35 people each:

(a) Group without cognitive load watching traditional commercials.
(b) Group without cognitive load watching material containing product placement.
(c) Group with cognitive load watching traditional commercials.
(d) Group with cognitive load watching material containing product placement.

#### Procedure

Participants in the first and the second groups were asked to watch a 4-min clip which, depending on the subgroup to which a given individual was assigned to, contained either traditional commercials or product placement (film material one and two). After watching the material, participants were given a board with 44 logos, from among which they were supposed to select four brands they recalled from the material and transfer to the response sheet.

The procedure in the third and the fourth groups accounted for introduction of cognitive load among participants. The film material presented to participants in that group (film material three and four) displayed instructions for simple mathematical tasks in the lower-right corner. The participants were asked to pay attention to the tasks and to perform simple operations in their heads, until an instruction appeared to write the result down; participants then recorded the result in one of three spaces designated for doing so. The instruction to record the results was intended to make participants more determined to closely follow the tasks and to keep their eyes trained on the lower-right corner. The background to the mathematical tasks was, however, the very same material as before – product placement or traditional commercials, depending on the subgroup to which participants were assigned. Three series of operations concluded simultaneously with the end of the film material. The participants then received the same board used in the control studies, and the rest of the procedure was analogical to the control group – recording recalled brands and defining consumer preferences.

### Results

Firstly, we examined the influence of sex on correct recall of companies’ logos – with regard to the fact that sex did not differentiate the results [*t* = 1.068 (138); *p* = 0.287, *eta*^2^ = 0.008], it was omitted from further analyses.

We examined the impact of the type of advertisement and cognitive load on accuracy in recalling the advertisement. Both the advert type (*F* = 14.05; *df* 1, 136; *p* < 0.001, *eta*^2^ = 0.094) and presence of cognitive load (*F* = 286.13; *df* 1, 136; *p* < 0.001, *eta*^2^ = 0.678) turned out to have a significant impact on the results generated. Recall of brands was generally better in conditions of traditional commercial than product placement, and better in neutral conditions than in conditions of cognitive load. From the perspective of the dependencies we examined in the study, of particular importance is that the interaction between the variables was statistically significant (*F* = 6.41; *df* 1, 136; *p* < 0.012, *eta*^2^ = 0.045). Yet here it is of key importance to compare the average number of brands remembered by participants in conditions of product placement in a neutral situation vs. traditional commercial in conditions of cognitive load. Considering the significant differences in variances of results in both groups, we applied a *t*-test with equal variances not assumed. It turned out that these differences were statistically significant: [*t* = 8.57 (40.57); *p* < 0.001; *eta*^2^ = 0.519], indicating better retention of brands in conditions of product placement.

**FIGURE 1:**
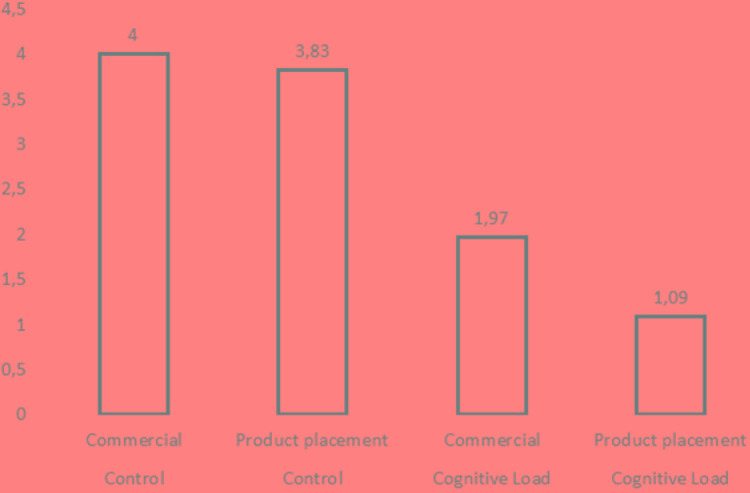
Average number of correct commercial recalls by group.

Averages and standard deviations in particular subgroups are shown in **Table 1**. To illustrate the obtained results better, the average values for particular experimental groups have been presented in the form of a graph (**Figure 1**).

**Table 1:** Number of correct commercial recalls by group.

Research condition	Type of advert	*M*	*SD*	*N*
Control	Commercial	4.00	0	35
	Product placement	3.83	0.382	35
	General	3.91	0.282	70
Cognitive load	Commercial	1.97	1.224	35
	Product placement	1.09	1.067	35
	General	1.53	1.224	70
General	Commercial	2.99	1.335	70
	Product placement	2.46	1.594	70
	General	2.72	1.489	140


## Discussion

The results we recorded demonstrate that in laboratory conditions people generally remember content presented in traditional commercials better than content promoted by way of product placement. The results also demonstrated that the mere induction of cognitive load among study participants significantly decreased their capacity to correctly remember the brands presented on the screen (this effect was particularly strong in respect of product placement – that group of participants recorded the lowest average results for correct recall of brands).

Conditions for watching traditional commercials and product placement adverts in real-life situations are, however, usually diametrically opposite. Product placement is a mechanism in which the advertised brand or product is naturally inserted into a film, entertainment program, news presentation or other program the viewer’s focus is trained on. In the case of the traditional commercial, the viewer is first informed of the upcoming advertisement, and then trains focus on something other than the contents of the commercial (e.g., engaging in an entirely different activity at the same time). In psychological research designed to have practical implications, it is very important to create conditions for a study which can be directly applied to real situations (see e.g., Anderson and Simester, 2004; Dholakia, 2006; Grzyb, 2016). By the same token, it should be considered that the proper test comparing effectiveness of the traditional commercial and product placement advertising in our experiment is comparison of the number of brands remembered by participants in the condition “traditional commercial and cognitive load” with “product placement and neutral situation.” As it turned out, retention of brands was higher in the latter of the two cases.

It is our opinion that this allows for the view that product placement is a valuable and effective marketing technique. The benefits of applying product placement are not limited only to the fact that the viewer’s attention is not drawn to other activities. Another highly beneficial aspect is that product placement relatively easily allows for reaching clients of specific demographic parameters with precisely defined interests and characteristics (considering the target group which a given film or other program is aimed at). The product placement method doubtlessly makes it possible to profile viewers, as prior to engaging in cooperation with the creators of a film the advertiser can be quite confident as to the traits of viewers that will be reached. Advertisers also enjoy influence over the manner in which their product will be shown in the program. A good example of this is the Ford brand, which exerts significant influence over the placements it purchases, giving filmmakers very firm instructions to ensure their cars do not appear in scenes involving “damaged brakes, drained batteries or punctured tires” (Turcotte, 1995). Product placement also allows for the use of the best technologies, including such elements as sound, color, and movement, something difficult to do in the case of other marketing tools (Corniani, 2001).

Naturally, when we think of the support that psychology (particularly social psychology) could provide to marketing and advertising, we do not limit ourselves only to research methodology. It has been demonstrated in many experiments that certain truths from psychological theories are an excellent fit in the practice of marketing. For example, Codaccioni and Tafani (2011) demonstrated how conversion theory (Moscovici, 1980) impacts the manifest and latent level of recalling advertising. While Codaccioni and Tafani did not explore product placement in their studies, they did draw attention to less-obvious ways of advertising products and more subtle measures used in checking recall of the content of persuasive messages.

Product placement is not, obviously, a trouble-free tool. One of the primary reservations concerning the method is the difficulty in advertising new products and ones just reaching the market. Products advertised in this manner are usually on screen for a few seconds; their location is rarely central, which makes it impossible to demonstrate all of the product’s attributes and functions, which is key from the perspective of advertising a product not familiar to the broader public (Corniani, 2001). Another weakness of this method is the absence of the possibility to predict the success of the production in which advertisers place their products. From the perspective of the phenomenon known as transfer of affect, described in more detail in the theoretical introduction, one may expect that a film which performs disastrously and receives poor reviews from critics can *de facto* damage the advertised brand and reinforce negative associations with the product (Redker et al., 2013). Lastly, although the study’s results indicate rather high acceptance on the part of viewers for this method (estimated at approximately 40%), one of the more serious flaws in product placement is growing criticism by viewers resulting from saturation of such messages in film productions (Narang, 2012).

Although the structure of the results we have attained allows for adopting the aforementioned conclusion as to the effectiveness of product placement, we feel obliged to point out several limitations in our study. Firstly, we examined only students, which render generalization of the results across all social groups invalid. For example, it cannot be excluded that significantly older individuals experiencing cognitive deficits are focused exclusively on key issues in the story, and neither notice nor retain brands of products that appear on the screen. Of course, it is only possible to resolve these issues by conducting more studies. It appears that it would be particularly important to carry out similar studies for various age groups – e.g., elderly subjects coping with natural, aging-related cognitive deficits. It would be crucial in such a case to appropriately select the stimuli, allowing for the natural preferences of the subjects. This factor was not included in our study, while it could affect remembering the content incorporated into product placement (e.g., individuals who had preferred one existing brand over another one might be more susceptible to product placement related to the former).

Secondly, a particular limitation of the presented study is the particular operationalization of the stimulus – cognitive load was induced in the form of simple mathematical tasks, appearing in the lower-right corner of the film material. Although this is a practice frequently applied in experimental studies exploring cognitive load (Chow and Luk, 2006; Elder and Krishna, 2010; Yoon et al., 2011), on the one hand, it may cause stress and discomfort among participants; on the other, it is somewhat artificial. As Pantoja et al. (2016) suggest, the application of common subtitles in a film is sufficient to induce cognitive load, even when the titles are in a language the participant knows fluently. Additionally, it must not be ignored that asking the subjects to tackle a mathematical task alone may cause increased fear of being evaluated (Rosenthal, 2009). The subjects may be asking themselves the following questions: did I do well, did I meet the expectations of the experimentators, and by the same token focus more on the correct mathematical result and less on their contact with the film stimulus. In future studies it would thus be worth examining whether the use of subtitles rather than mathematical tasks would generate similar statistical results while also making participation in the procedure easier. Thirdly, the results we have received only apply to one of many indicators of marketing activity success. Indeed, we examined retention of brands by participants. The question arises as to the relative (e.g., compared to conventional commercial) effectiveness of product placement in reference to other issues, and particularly its influence on the purchase by viewers of particular brands. These issues should also be settled in future empirical studies.

## Author Contributions

TG and DD were equally responsible for designing the experiment, analyzing results, and writing article. AK was responsible for conducting the research and obtaining results.

## Conflict of Interest Statement

The authors declare that the research was conducted in the absence of any commercial or financial relationships that could be construed as a potential conflict of interest.
